# Treatment of Puerperal Septicæmia

**Published:** 1884-03

**Authors:** 


### Selections.

### TREATMENT OF PUERPERAL SEPTICÆMIA.

Let us now suppose that, in spite of every precaution, the specific poison has gained entrance at one of the numerous doorways left open in the genital tract between the vulva and the fundus uteri; what are the most reliable means now known to us for checking the advance of the septic disorders which are set up in consequence?

But let me stop here, before answering the question just asked, and explain what I mean by the use of the term specific poison. I do not believe that there is, necessarily, any specific disease germ which gives rise to *puerperal* septicæmia! It is probably the same germ as that which is the source of septicæmia, phlebitis, and lymphangitis in the stump after an amputation, in the wound created by a compound fracture, or in the lacerated tract produced by a gun-shot. But the pathological condition excited appears to me to be entirely different from that putrid absorption which results from the decomposition of a retained placenta, or a putrid mass of blood. Such decomposition produces a toxæmia, violent and dangerous it is true, but which disappears as soon as the offending mass is removed. That of the true puerperal disease at once, or almost at once, diseases the lymphatics and sets up an action which often proves uncontrollable. If the mere presence of decaying animal material in a uterus would produce puerperal septicæmia without the agency of a specific disease germ, we should surely have that affection developing in healthy country localities where the women are attended by ignorant midwives, but where, nevertheless, it is almost an unknown disorder.

"I," says Hervieux, "who write these lines, declare that in my own country I have within the space of three years attended one thousand cases of labor, and out of that number have lost only one patient!"

And now, in summing up what I esteem the most certain and the most rational treatment of the disease styled puerperal fever, I will be as concise as possible.

As soon as the patient is stricken by the poison, certain very marked phenomena usually develop themselves with great promptness. After a chill or a slight horripilation she is affected by a high temperature, pelvic pain, considerable mental perturbation, headache, pain in the back, and sometimes, though not commonly, by nausea and vomiting. We will assume, first, that the attack is a severe one in its inception; and, second, that the patient is in such a position in life that we are not in any way hampered in our efforts to save her by considerations of economy. Having considered treatment from these standpoints, it will, of course, be easy to modify the plan so as to meet the requirements of a mild attack or of a scanty purse.

As the practitioner sits by the bedside of his patient at the commencement of her attack, he is aware that there are points connected with its true pathology which he cannot yet determine. For example, he cannot say whether the case is going to assume the form of septicæmia lymphatica or septicæmia venosa; whether of perimetritis or parametritis; or whether thrombosis of some of the large pelvic or utero-ovarian vessels, or a true parenchymatous metritis is to play the most active part in the siege which has begun. If he fritter away the golden moments in vague speculation; if he soothe his fears by hoping that the attack is due to malaria or milk fever; or, if he cast aside the rational doctrines of to-day in favor of the idea of a general infectious and particular form of disease called by the forefathers of the French school, "*la fievre des femmes en couche*," time will be lost which can never be regained. If, on the other hand, he is encouraged by his clinical observation to stand with many of the best pathologists and practitioners of our time in the position assumed by Hervieux—"I believe in the multiplicity of the affections classed under the head of puerperal fever; I believe in puerperal poisoning as the source of them"—he will act at once and strike at the poison before it has fairly gained a foothold. In other words, if the physician could see into the future and learn with certainty that peritonitis, cellulitis, thrombosis, lymphangitis, or true phlebitis is to be the final disorder, he should, if he reaches the case at the inception of the attack, follow, in my opinion, the course here formulated:

1. As soon as a diagnosis of septicæmia is determined upon, all pain, nervous perturbation, shock, and mental anxiety should be quieted by the hypodermic administration of ten minims of Magendie's solution of morphine, unless some special and very decided idiosyncrasy with reference to opium be ascertained to exist; and throughout the severity of the attack, whenever suffering of mind or body occurs (perhaps it will be about once in every six or eight hours), this should be repeated. In my experience, no other method of administering morphine in these particular cases compares with this, and, as it is not to be continued long, there is no fear of causing the patient to become addicted to the drug as a vice. If a small, sharp, and new needle be used, if it be thoroughly cleansed with soap and water before each time of using, and be dipped in a solution of bichloride, 1 to 1,000 of water, just before each insertion, no abscesses will occur. It is the large, rusty, unwashed and unpurified needle which the doctor's economy makes last him for many months, which so commonly results in them.

2. The physician must now decide whether, in his opinion, the septic disease which is developing has originated in the wounds situated between the os internum uteri and the vulva, or in the endometrium, above the former point. If he decide in favor of the former view, he should persist, for a time longer, in the more thorough use of vaginal injections; if of the latter, intra-uterine injections should be at once resorted to. Usually the question has to be decided by the efficacy or inefficacy of frequent germicide vaginal injections in bringing down the temperature and controlling other grave symptoms. Should the failure of these seem to prove that the origin of the disease is higher up the genital tract, more decided and radical measures must be taken.

The patient having been entirely relieved of pain and thoroughly quieted, the first injection should be practiced in this way: An Indian rubber cloth should quietly, without hurry, noise, or disturbance on the part of the nurse, be spread over the edge of the bed on which she lies, and made to fall into a tub of warm water rendered antiseptic by the addition of 2 or 2½ per cent. of carbolic acid, or of the bichloride of mercury, 1 to 2,000, or of some other reliable germicide. Then Chamberlain's glass uterine tube, which I here show, or the very excellent and ingenious tube invented by Dr. George H. Lyman, which is here seen, thoroughly fitted to a Davidson's or Higginson's syringe, should be immersed in the tub. The nurse now aiding the patient by the shoulders, and the doctor by the hips, she should be gently laid across the bed and be made comfortable with a pillow under the head. Each foot should rest upon a chair placed at either side of the tub, and she should be entirely covered over with a couple of blankets. The doctor, now placing himself between the knees of the patient, should take the tube in his right hand while a stream of water is made to flow through it by the nurse, who squeezes the syringe bulb, and he should pass it gently up the fundus of the uterus. The stream of water, which has been steadily flowing, is now projected with gentle force against the walls of the uterus, washing away adherent blood-clots, detaching portions of hanging membrane, and everywhere neutralizing the influence of the poison which has excited the disorder.

After the first injection, the position of the patient need not be disturbed, but the injections may be given as she lies upon a bed-pan.

In some cases, in which I have had reason to suspect that portions of the placenta or membranes have been retained, I have chloroformed the patient, passed the hand, rendered thoroughly aseptic, within the cavity, and very gently scraped off adherent masses from the uterine walls, using the nails as a curette, as Wilson, of Baltimore, has advised. In some other cases I have rubbed the whole endometrium with an aseptic sponge, held in a long sponge-holder, or employed the largest of my curettes to remove clots and adherent secundines, with great apparent advantage.

That the use of antiseptic uterine injections after parturition is attended by danger is beyond question. The greatest hazard attending this plan is the entrance of air into the uterine cavity; the next, the production of hemorrhage by detaching some of the thrombi which fill the mouths of the uterine sinuses; the third, the danger of forcing the fluid used as an antiseptic directly into the general circulation, through the introduction of the tube into the mouth of a sinus; fourth, the creation of convulsions, violent pain, or nervous prostration, by a sudden and baneful influence upon the nervous system; and the fifth, the passage of the tube into a Fallopian canal, and the injection of fluid directly into the peritoneal cavity, as in a case reported by Dr. W. Gill Wylie in an interesting paper in the *New York Medical Journal* for June 23, 1883, p. 679.

All these dangers may be, to a great extent, avoided by care as to details, by using a large injecting tube which cannot enter an open-mouthed sinus; by using water warmed to 105°; by injecting the fluid through the tube so as to exclude air before passing this up to the os uteri; by using only a moderate degree of force in throwing the jet against the uterine walls; and by proceeding with the whole affair gently, cautiously, slowly, and intelligently.

The tube should never be allowed to fill the os uteri completely, so as to prevent the escape of the injected fluid. Should the cervical canal be so narrow as to hug the tube closely, it should be dilated by dilators of hard rubber, by the fingers, or Barnes' bags, before the injection is practiced.

A solution of the persulphate of iron should always be at hand in case of sudden hemorrhage from displacement of a thrombus. Should this accident occur, ergot should be immediately given hypodermically, the iron solution be at once added to the antiseptic solution and allowed to pass into the uterus, and pressure be made upon the fundus so as to stimulate the contraction of uterine fiber to accomplish closure of the open sinuses in that way.

Quite a number of cases of death from this plan of treatment are on record. In a very large experience with it I have met with but one. The whole number on record would, however, fall, I think, into insignificance if weighed in the balance against the many deaths which have been due to a neglect of the means, or against those lives which have been saved by it.

After all, the question as to the dangers attending a plan of treatment are not to be settled upon mere abstract reasoning. The evil which it is known to do must be weighed in one scale, and the good which it effects in another; and careful consideration must decide whether we are justified in accepting the former for the sake of the latter. Judged in this manner, I feel very sure that intra-uterine injections for perperal septicæmia deserve a place among the most valuable resources for the saving of life for which we are indebted to modern pathology.

The frequency of these intra-uterine injections should vary greatly with individual cases. In mild cases of septicæmia, where the temperature comes readily down after the uterus has been washed out, and rises very slowly, they need only be used once in every five hours; in other cases they become necessary once in every three hours; and in bad cases they are required once every hour. These injections should always be administered by a physician, should always be carried fully up to the fundus uteri and should always be used with every regard to caution as to detail which has been already mentioned.

Many prefer the use of those syringes which allow of a steady flow of a stream of water propelled by gravitation, as is the case with the so-called fountain syringe, which is so popular among us. This is partly because greater safety is supposed to attach to these, and partly from a theory that danger attends the propulsion of a stream by intermittent jet against the uterine walls. For a number of years I shared this belief, but experience has taught me that a gentle projection of the fluid is an advantage, that by this means a more thorough cleansing is accomplished, and that with due caution no more danger attends the plan than that by the steady flow.

Some have adopted continuous irrigation of the uterine cavity, but this is, I feel perfectly certain, a delusion and a snare. It gives the appearance of great thoroughness, which it does not possess, for the reason that by this plan it is very difficult to bring the germicide fluid into full contact with the entire endometrium. For vaginal irrigation it is an excellent method, but I have seen it allow the temperature to remain high when applied to the uterine cavity, and have replaced it by the intermittent douche, used only as often as every three hours, with striking results. Nevertheless, in very severe cases I prefer to employ continuous irrigation, replacing its use every third hour by that of the intermittent current; rather than exhaust my patient by half-hour disturbances and injections, as has been by some advised.

After all that has been said on this subject, the essential fact is this: that plan is best which accomplishes most perfectly the cleansing of the parturient canal. With ordinary precautions, danger need not necessarily attach to any method.

3. The uterus having now been thoroughly cleansed, and the patient entirely quieted, attention should be turned to controlling the temperature, which in septicæmia of puerperal character, runs so high and maintains itself at so exalted a range as to constitute one of the immediate factors of a fatal issue. Even if this were not the case, the patient's strength is so much exhausted by prolonged high temperature, her nerve powers so much depreciated, her blood-state so rapidly injured, and her comfort so decidedly interfered with, that these considerations alone would point to the propriety of combating hyperpyrexia. For this purpose I formerly relied upon the affusion of cold or tepid water, the patient lying upon Kibbee's cot; at present I accomplish the same result more easily and more pleasantly for the patient by the use of Chamberlain's rubber tube coil, which I here show. A mat, composed of a rubber tube rolled upon itself in a circle, covers the whole abdomen from the ensiform cartilage to the symphysis pubis; the upper end of the tube which makes this mat is anchored by a weight in a tub of ice-water, placed about three feet above the level of the patient, and the lower end falls into a tub upon the flour. By siphon action the water of the elevated tub runs through the tube which constitutes the mat, and collects in the receptacle on the floor. By this means a temperature of 104° can very readily, as a rule, for there are exceptions to the rule, be kept at 100° for weeks together.

Unfortunately, there are almost insurmountable difficulties connected with the use of this invaluable method in the minds of the patient's friends, the patient herself, the nurse, and alas, too often, the attending physician. You are told that the patient becomes chilled, that the coil prevents her resting, that the temperature absolutely goes up under its use and descends whenever it is left off. By the doctor you are apt to be informed that his fear of resulting pleurisy, bronchitis or pneumonia is very great.

I will merely say, in refutation to these charges, that in my service in the Woman's Hospital, where convalescents from laparotomy are constantly under treatment in large numbers, this means of controlling temperature is as commonly and as freely in use as poultices are in general hospitals, or gargles in dispensaries for diseases of the throat. We never meet with any of these difficulties, and very rarely with failures as to the desired result, and I believe that I am correct in saying that successive house-staffs whose duty it has been to carry out the plan have thus far had, to a man, the most implicit faith in its beneficial agency.

There are some peculiarities about it, however, which I must mention: Very often the coil will not succeed in controlling the temperature for twenty-four hours; its prolonged use alone develops and illustrates its great benefits; and removing it from the body for an hour at a time damages its influence very much. I have never seen evil result from the chilliness which it excites, if hot bottles be kept at the soles of the feet, and in not one instance out of hundreds of cases have I seen pneumonia or pleurisy excited by it.

4. The nervous system should be kept under the influence of febrifuge medicines as to keep under the control the tendency to chill and pyrexia. For this purpose, fifteen grains of sulphate of quinine should be given in capsule or by suppository night and morning, or, in place of this, two capsules may be given night and morning of Warburg's tincture, in the form of solid extract, as advised by Dr. J. T. Metcalfe. Lastly, to the same end the salicylate of sodium may be employed.

5. The patient's diet should consist entirely of fluid food, given often and in small amounts. The staple article should be milk, but animal broths and gruels may be alternated with it with advantage.

6. At the very commencement of such a case the attending physician should, in the patient's interest, surround himself with efficient and abundant assistance. If he undertake to wash out the uterus every four or five hours without other assistance than that of the nurse; and if the patient is to rely for the constant attention and care which she will inevitably require, upon one nurse, it is needless to point out that the duty of both doctor and nurse—vital duties, be it remembered—will be but half performed. And, unfortunately, the penalty will fall upon the patient and her friends.

For a case of puerperal septicæmia to be properly treated in private practice by the plan here advocated, it is necessary for the attending physician to associate with himself some young practitioner, who has the time to devote to the case, and the intelligence to use the uterine douche safely and efficiently. Furthermore, two nurses are necessary; one to be in charge for twelve hours, and the other then to relieve and replace her for an equal time. Without the rest and sleep thus afforded, no nurse taking charge of such exacting cases as these can possibly do her duty in such a way as to subserve the patient's interest. As a rule, the nurse will begin with the declaration that she is competent and willing to take entire charge; that she is one of those people who can do without sleep, etc.; and her heroic offers of self-sacrifice are hailed with enthusiasm by the terrified family. To believe in and to act upon this would be very foolish and very dangerous. After three nights of watching, this same nurse would snore through the hours in which her patient needed her, give the wrong medicines, mislead the doctor, allow the bladder to become distended with urine, and fail in every requirement of the occasion.

If the patient cannot bear the great expense attendant upon the extra service which I have mentioned, it is her misfortune, and for such as herself the doors of our hospitals are open. There is no case of ovariotomy in the Woman's Hospital, however poor the patient be, upon which, thanks to the generous arrangements of its managers, just such attendance as I have mentioned is not lavished.

The antiseptics which have heretofore been tried under these circumstances are thymol, boric acid, salicylic acid, carbolic acid, and mercuric bichloride. Of these, all have disappeared before the superior merits of the last two; and carbolic acid, which for so long a time has been almost supreme, appears about to be abolished in favor of the bichloride of mercury, 1 part to 1,000 or 2,000 of water. For all antiseptic purposes outside of the uterus, the bichloride is now, owing to the carefully made and important investigations of Koch, very generally employed in the strength of 1 to 1,000, and the uterus has now been washed out with this excellent germicide 1 to 2,000 often enough to make us regard its use as an intra-uterine injection as entirely warrantable. If carbolic acid be used in that way, it will not be safe to carry its strength beyond a two, or, at most, a two-and-a-half per cent. solution.

